# Mind Wandering Influences EEG Signal in Complex Multimodal Environments

**DOI:** 10.3389/fnrgo.2021.625343

**Published:** 2021-04-01

**Authors:** Jonas Gouraud, Arnaud Delorme, Bruno Berberian

**Affiliations:** ^1^Systems Control and Flight Dynamics Department, Office National d'Etudes et de Recherche Aérospatiales, Salon de Provence, France; ^2^Center of Research on Brain and Cognition (UMR 5549), Centre National de Recherche Scientifique, Toulouse, France

**Keywords:** out of the loop, mind wandering, automation, vigilance, attentional decoupling, sensory modalities

## Abstract

The phenomenon of mind wandering (MW), as a family of experiences related to internally directed cognition, heavily influences vigilance evolution. In particular, humans in teleoperations monitoring partially automated fleet before assuming manual control whenever necessary may see their attention drift due to internal sources; as such, it could play an important role in the emergence of out-of-the-loop (OOTL) situations and associated performance problems. To follow, quantify, and mitigate this phenomenon, electroencephalogram (EEG) systems already demonstrated robust results. As MW creates an attentional decoupling, both ERPs and brain oscillations are impacted. However, the factors influencing these markers in complex environments are still not fully understood. In this paper, we specifically addressed the possibility of gradual emergence of attentional decoupling and the differences created by the sensory modality used to convey targets. Eighteen participants were asked to (1) supervise an automated drone performing an obstacle avoidance task (visual task) and (2) respond to infrequent beeps as fast as possible (auditory task). We measured event-related potentials and alpha waves through EEG. We also added a 40-Hz amplitude modulated brown noise to evoke steady-state auditory response (ASSR). Reported MW episodes were categorized between task-related and task-unrelated episodes. We found that N1 ERP component elicited by beeps had lower amplitude during task-unrelated MW, whereas P3 component had higher amplitude during task-related MW, compared with other attentional states. Focusing on parieto-occipital regions, alpha-wave activity was higher during task-unrelated MW compared with others. These results support the decoupling hypothesis for task-unrelated MW but not task-related MW, highlighting possible variations in the “depth” of decoupling depending on MW episodes. Finally, we found no influence of attentional states on ASSR amplitude. We discuss possible reasons explaining why. Results underline both the ability of EEG to track and study MW in laboratory tasks mimicking ecological environments, as well as the complex influence of perceptual decoupling on operators' behavior and, in particular, EEG measures.

## Introduction

### Context

The last decade has seen important research toward road automation (Badue et al., 2019). Promised as a revolution for users to gain flexibility, leisure time, and safety (Harb et al., 2018; Correia et al., 2019), self-driving cars have nonetheless several important technological gaps that must be filled before becoming a reality. On the way toward level 5 automation (full automation anywhere, see SAE International, 2018), teleoperation could represent an important trade-off to maintain safety while developing system capabilities. Teleoperation, literally operating a vehicle at a distance, is already used in environments unreachable or dangerous to humans, such as war theaters, nuclear environments, and space (Lichiardopol, 2007). Tomorrow, teleoperation could be performed by algorithms in the cloud and allow any vehicle to reach level 5 automation with minimal modifications (Zhang, 2020). However, the technology could also use human intervention today to enhance partial automation and widen its operational design domain (Kang et al., 2018). Operators would then monitor a set of vehicles, taking control whenever necessary, such as in the event of snow or in an emergency. Specifically, an important advantage of human teleoperation is the assumption that there could be more vehicles to monitor than operators, as not all vehicles would require assistance at the same time (Zhang, 2020).

Aside from technical challenges like latency (Neumeier et al., 2019), the possibility of jumping into a specific situation only when needed raises important interrogations regarding the ability of operators to assume manual control when needed. Humans would then only have to monitor, presumably ever-alert, for deviations and problems. Situations where operators are supervising automated control loop are called out-of-the-loop (OOTL) situations (Norman and Orlady, 1988; Endsley and Kiris, 1995). Unfortunately, OOTL situations reduce the operators' ability to intervene, if necessary, and to assume manual control, i.e., to come back in the control loop (Kurihashi et al., 2015; Louw et al., 2015a; Naujoks et al., 2016). Supervisors at this point seem dramatically powerless to diagnose the situation, determine the appropriate solution, and execute it before the accident happens. Accident reports may contain the terms “total confusion” (National Transportation Safety Board, 1975, 17; Bureau d'Enquête et d'Analyse, 2002, 167), “surprise effect” (Bureau d'Enquête et d'Analyse, 2012a, 44, 2016, 10), or “no awareness of the current mode of the system” (Bureau d'Enquête et d'Analyse, 2012b, 178). These negative side effects on overall performance are commonly referred to as OOTL performance problems.

Nowadays, it is assumed that OOTL performance problem is fundamentally a matter of human–automation interaction arising from both operators' internal states and system properties, which ultimately spoils performance (Berberian et al., 2017). From this definition, one way to mitigate related performance drops may be to monitor operators' internal states and look for precursors to OOTL performance problems. Among others, it has been demonstrated that non-challenging tasks, such as passive monitoring of automation, can promote episodes of mind wandering, whereby attention drifts away from the task at hand (Smallwood et al., 2008; Durantin et al., 2015; Smallwood and Schooler, 2015; Gouraud et al., 2018a,b; Dehais et al., 2020). Mind wandering (MW) is a family of experiences unrelated to the here and now (Seli et al., 2018). When MW happens during a task, it moves operators' minds away from their tasks toward matters not directly related to their current works. Although such uncontrolled thoughts could be beneficial as long-term planning and mind refreshment (McMillan et al., 2013; Ottaviani and Couyoumdjian, 2013; Terhune et al., 2017), it may thwart short-term performances (He et al., 2011; Galera et al., 2012; Cowley, 2013; Casner and Schooler, 2014; Dündar, 2015). Therefore, real-time tracking of MW is an important goal within safety-critical industries, particularly when automation supervision fills a significant part of the job. Indeed, real-time tracking of internal states like MW would allow detecting problems before performance drops and accidents happen. However, a better understanding of the emergence of this attentional decoupling remains essential to achieve such a goal. This is precisely the objective of this study.

### Emergence of Attentional Decoupling

Many physiological tools have already demonstrated sensitivity to several aspects of MW; however, electroencephalography (EEG) is among the most promising. EEG signal has already helped uncover an important facet of MW: attentional decoupling. People subject to MW experience a drop in the cortical processing of the external environment, as their attention is redirected to inner thoughts (Schooler et al., 2011). Neurologically, attentional decoupling is characterized by weaker neuronal responses to external stimuli and greater deactivation of the regions dedicated to their processing. During GO/NOGO tasks, researchers (Kam et al., 2011) showed that the amplitude of P1, N1, and P3 components (respectively associated with visual perception, auditory perception, and external stimuli processing) were all lower during task-unrelated MW. This effect held true whether stimuli were the SART (Sustained Attention to Response Task) stimuli or irrelevant to the task. Such results were replicated in two other settings: a time-estimation task (Kam et al., 2012) and during monotonous manual driving (Baldwin et al., 2017). It was also highlighted through ERPs that attentional decoupling involved lower emotional reactions (Kam et al., 2014). Experiments also uncovered the signature of MW on alpha waves in occipital, i.e., visual stimuli processing, areas (O'Connell et al., 2009; Braboszcz and Delorme, 2011; Baird et al., 2014; Atchley et al., 2017; Baldwin et al., 2017; Arnau et al., 2020), although the exact way is still debated as explicated in the next sections. Nevertheless, changes in alpha activity during MW are in line with the alpha band being involved in the deactivation of the concerned areas (Bonnefond and Jensen, 2012; Benedek et al., 2014; Villena-González et al., 2016).

### Factors Influencing Attentional Decoupling

Even though MW has a strong influence on the neuronal signal, the factors modulating the attentional decoupling remain unidentified. A first important question is the exact degree of attentional decoupling. Put differently, do all MW have the same potential for attentional decoupling? Is “depth” a feature of MW episodes? Several studies provide insight into depth as a feature of MW episodes. Cheyne et al. (2009) used a SART to investigate the validity of their bi-directional model of inattention. They obtained converging measures supporting three postulated states of inattention: level 1 characterized by more erratic reaction time, level 2 by anticipations, and level 3 by omissions. Following the same path, Schad et al. (2012) detailed the “levels of inattention hypothesis” based on the assumption that our mind processes information sequentially, involving greater complexity at each step. MW could then thwart information processing at different stages, depending on the depth of the episode. While some MW episodes could be superficial, only impacting higher cognition, others could completely decouple from the task by blocking external information encoding and “cascade through the cognitive system” to impact more complex processing (Smallwood, 2011).

A second issue refers to the impact of MW on non-relevant stimulation. It was initially assumed that MW involves a specific impairment in the processing of task-relevant events (e.g., Smallwood et al., 2003, 2004). Studies using ERPs have shown that MW dampens the processing of sensory information, regardless of the relevance of this information to the task (Barron et al., 2011; Kam et al., 2011). However, the fact that MW can impact mechanisms of selective attention does not mean that all stages of sensory processing are turned off. Rather, it signifies that the highlighting of specific sensory inputs for higher levels of cognitive analysis is attenuated. After all, we are able to perform most of our daily tasks without any errors, even during MW episodes. In this context, steady-state responses (SSR) may highlight the exact impact of MW on cognition. An SSR is an evoked potential emerging from external periodical stimulus and whose phase and amplitude remain constant (Picton et al., 2003). Multiple studies have highlighted that in environments with multiple SSR competing for attention, focusing on one SSR increases its amplitude to the detriment of the others (Skosnik et al., 2007; Müller et al., 2009; Saupe et al., 2009a; Diesch et al., 2012; Mahajan et al., 2014). However, it has been shown that this effect is highly dependent on experiments' features: paying attention to a 20-Hz ASSR presented on one ear showed increase amplitude ipsilaterally, but not for a 40-Hz stimulus (Müller et al., 2009); in another study, the attention-competition effect decreased SSR amplitude only when concurrent SSR were presented on the same sensory modality (Porcu et al., 2014). These results highlight the complexity of the different stages of perception and attention, and SSR may help to understand the influence of MW on them. Moreover, if SSR were to be impacted by MW, it could reveal extraordinarily useful to study the features of attentional decoupling. Indeed, it would allow continuous monitoring, contrary to ERPs, while being fully controlled in frequency, in contrast to natural brain waves. O'Connell et al. (2009) has already investigated the influence of lapses of attention on a visual SSR without finding significant results regarding its amplitude. However, they did not use a questionnaire to track MW episodes. To our knowledge, no research has specifically addressed the impact of internally directed attention on SSR amplitude.

Our purpose in this experiment is to evaluate the viability of MW neuronal markers in complex laboratory tasks mimicking automated ecological environments, as well as help to characterize features of the attentional decoupling in these environments. Our hypotheses are (1) the evolution of MW can be tracked in complex environments through a decrease in ERPs and ASSR amplitude coupled with an increase in alpha power during MW episodes compared with focus moments, (2) MW attentional decoupling demonstrates a gradual nature on EEG measures (ERP, alpha, ASSR) correlated to the proximity of the thoughts content to the task at hand; more precisely, a MW episode with thoughts closer to the immediate environment will have less influence on EEG measures than another MW episodes with thoughts totally unrelated to the here and now.

## Materials and Methods

### Participants

We performed an a priori analysis to estimate the required sample size. Most publications investigating the links between MW and EEG did not report effect size explicitly. However, as repeated-measure ANOVA was often used, we could calculate from these publications Cohen's *f* using F-value, CI, and degrees of freedom. The lower value computed, which we retained to adopt a conservative view, was 0.54 (Kelley, 2007a,b, 2020; Uanhoro, 2017). We then used G^*^Power (Faul et al., 2007, 2009) to calculate the sample size, which yielded a minimum of 14 participants.

Eighteen participants (12 females, all right-handed) performed the experiment (age ranging from 21 to 45 years; *M* = 25, *95% CI* = [22; 29]). After pre-processing the data, we discarded three subjects:

- one subject reported “external distraction” on half experience-sampling probes (see Experience-sampling probes);
- a second subject reported 85% “task-related MW” but only one “task-unrelated MW”; moreover, only one epoch linked to “focus” was free from artifacts (two out of three epochs were discarded due to muscle activity). Subsequent questions at the end of the experiment revealed that he/she did only partially understood the difference between task-related MW and task-unrelated MW;
- a third subject displayed many movements during the experiment (foot tapping, jaw clench, arm movements), which were later found heavily decreasing data quality.

This resulted in 15 subjects in the analysis. The participants in this study were volunteers from the ONERA Company (ONERA, the French Aerospace Lab) or Marseille University. They received 20€ vouchers (cards for online payment) for the experiment. All the participants had normal or corrected-to-normal visual acuity and hearing, had no neurological or psychiatric disorders, and were not under any medication. All participants signed a written declaration of informed consent. The procedure was approved by ONERA ethical committee and was conducted in accordance with the World Medical Association Declaration of Helsinki.

### Experimental Tasks

#### Environment

**Figure 1:**
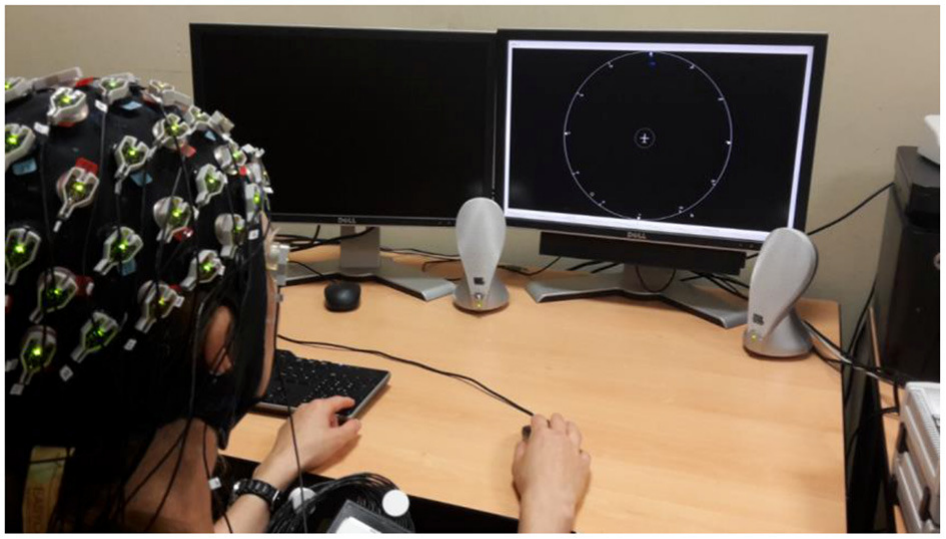
Experimental setup. The participant is equipped with the EEG system and sits in front of the right screen (LIPS screen). Speakers are on both sides of the right screen. The left screen is used to display attentional probes.

Participants were seated in front of a desk with two screens, two speakers, a keyboard, and a mouse (see Figure 1). Participants performed two tasks in parallel: a visual task and an auditory task. The visual task, an obstacle avoidance task (see Visual task), was displayed on the right screen. The auditory task was presented with speakers on the left and right sides of the right screen, which sent the beeps at semi-random intervals as well as the continuous modulated brown noise (see Auditory task). On the left screen, attentional probes appeared semi-randomly (see Experience-sampling probes).

#### Visual Task

The visual task consisted in the supervision of an obstacle avoidance simulator displayed on the right screen (the Laboratoire d'Interactions Pilote-Système (LIPS), or Pilot-System Interactions Laboratory an ONERA distributed simulation environment). The aircraft moved at a constant speed. It was displayed in white onto a 22″ LCD monitor (with a 1,024 × 768 pixel resolution and a 60-Hz refresh rate) located about 50 cm from the participant in an unlit room.

**Figure 2:**
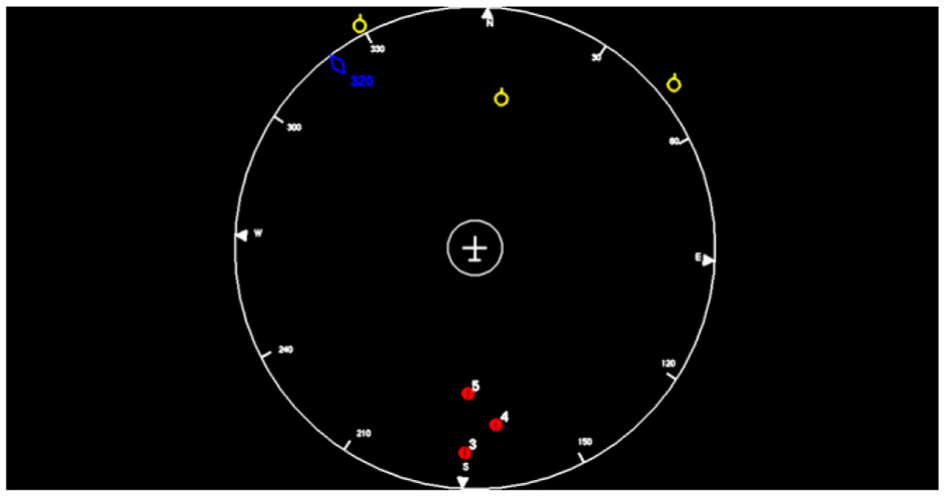
Screenshot of the LIPS interface. The plane in the center is static and the surrounding (yellow and red numbered symbols) are moving. During the left and right avoidance maneuver, again, the plane remains static and the background rotates.

The visual task displayed an unmanned air vehicle (UAV) depicted as a plane seen from above. The vehicle stayed at the center of a 2D radar screen (right screen, see Figure 2) and moved following waypoints arranged in a semi-straight line with clusters of obstacles along the way (every 45 s on average). Each cluster could contain between one and five obstacles, including one on the trajectory. When an obstacle was present on the trajectory (a situation called “conflict”), the autopilot detected it and initiated a left or right deviation, depending on the placement of the obstacles. Once the obstacle on the trajectory had been cleared, the UAV initiated another maneuver to come back on its initial straight-line trajectory. Participants were instructed to monitor the UAV, acknowledge its decisions, and correct any mistake the autopilot might make, i.e., choosing an avoidance trajectory that would result in an impact with another obstacle. In more details:

– Whenever they saw the autopilot changing the trajectory, participants clicked on an “*Acquittement*” (acknowledgment) button to acknowledge automated avoidance decisions (twice per conflict, once to acknowledge avoidance of the object and once to acknowledge the return to normal trajectory after avoiding the object);
– If they detected an incoming collision, they clicked on the button “*Changement d'altitude*” (change height) so that the UAV would perform an emergency descent to avoid colliding with the obstacle.

In both cases, a feedback message was displayed to the participants whenever they clicked.

#### Auditory Task

An auditory task was proposed at the same time as the visual task. Participants had to react as fast as possible to beeps (100 ms duration, 1,000 Hz frequency). Participants had 1 s to answer to these beeps presented at semi-random intervals; if they did not respond within the given time, the auditory stimulus was counted as a miss. This task was supported by E-Prime 2.0 (Psychology Software Tools, 2018). The auditory task was used to measure attention through reaction time and EEG measures (see Electroencephalography).

On top of the beeps for the auditory task, we played using E-Prime a background brown noise modulated in amplitude to elicit ASSR. Amplitude modulation was chosen as the most widely used steady-state stimuli (Picton et al., 2003) better tolerated by people than clicks (Voicikas et al., 2016). We first generated brown noise using the *acoustics.generator.brown* function (felipeacsi and Rietdijk, 2018). This signal was then modulated with a 50% and 40-Hz sinusoidal amplitude modulation. Because E-Prime loads file sounds as the experiment develops, a 1-h file would have exceeded the cache memory. To allow for easier loading, we divided the sound into 5-s soundtracks played one after the other in a loop (Supplementary Audios 1–3). To avoid participants to develop explicit or implicit learning with repetitive sound features, we generated three different 5-s soundtracks, which E-Prime played in random order. Tests before the experiment did not reveal any audible problem when switching between soundtracks, nor did participants realize it when asked after the experiment (Agus and Pressnitzer, 2013). We used Python 3.6 to generate modulated background brown noise with the base packages *acoustics, wave, math*, and *random* (Python Software Foundation, 2018).

#### Experience-Sampling Probes

On average, every 2 min, an experience-sampling probe programmed with E-Prime 2.0 (Psychology Software Tools, 2018) appeared on the left screen (Figure 1). For technical reasons, the visual task (obstacle-avoidance task) was not paused when the experience sampling probes appeared. Participants were asked to answer the probe as soon as it appeared, and any successful or failed trial on the obstacle-avoidance task during this interval was not taken into account to compute their performances on the visual task. Participants were informed that the questionnaire probes were for informational purposes only and were not used to assess performance.

Participants were required to answer the following question (originally in French): “When this questionnaire appeared, where was your attention directed?” Answers could be “On the task” (focused, e.g., thinking about the next obstacle, the decision to make, the incoming waypoint), “Something related to the task” (task-related MW, e.g., thinking about performance, interface items, last trial), “Something unrelated to the task” (task-unrelated MW, e.g., thinking about a memory, their last meal, or a body sensation) or “External distraction” (e.g., conversation, noise). The preceding examples were given to participants to illustrate each category before the experiment. We were primarily interested in reports of being focused or having task-related or task-unrelated MW. The possibility of reporting “task-related MW” was proposed to avoid participants reporting task-unrelated MW when thinking about their performance (Head and Helton, 2016). The answer “External distraction” was proposed to avoid participants reporting MW if they were distracted by a signal external to themselves and the task.

### Procedure

Sessions started with an explanation of the two tasks, followed by a 10-min training period and a 55-min session. During this study, participants had to perform the visual task (supervise the UAV avoiding obstacles and acknowledge or correct any mistake, see Visual task) and the auditory task (press a button as fast as possible when hearing a beep, see Auditory task) at the same time. The session contained 70 clusters of obstacles for a total of 210 obstacles. Clusters were separated by 45 s on average. All autopilot decisions and collisions were predefined and, therefore, they were the same for all subjects. The autopilot made two errors initially placed randomly (3% errors; errors on trials 31 and 52 for all subjects). This low error rate was chosen to have a relatively safe system and reproduce ecological OOTL conditions.

Parallel to the visual task, participants performed the auditory task and had to react to infrequent beeps by pushing “Enter” button as fast as possible with their left hand. This secondary task served as a way to measure attention (see Measures and analysis for the exact measures reported). They were explicitly told that beeps and experience-sampling probes were to be treated as fast as possible, whatever was happening on the obstacle-avoidance task. Beeps were presented every 20–40 s. On average, one out of three beeps was followed by an attentional probe. In total, 32 probes were displayed during the whole session. The distribution of the experience-sampling probes was not correlated with events on the obstacle-avoidance task, to minimize performance influence on experience-sampling reports. We instructed participants not to pay attention to the ASSR background sound.

### Measures and Analysis

We used R-Studio 1.1.456, R 3.5.1 (RStudio Team, 2015; R Core Team, 2016) for statistical analysis, and Matlab 2018a (The Mathworks Inc., 1992), EEGLAB (Delorme and Makeig, 2004), and FieldTrip (Oostenveld et al., 2010) to filter and analyze EEG data. All 95% CIs reported hereafter were computed using the *boot* R package with 10,000 iterations with normal bootstrap approximation (Canty and Ripley, 2017).

All linear mixed-effect analyses used the R *lme* function to create the models (Bates et al., 2017), with a random intercept for subjects to account for our repeated-measure design. Each time, we visually inspected residual plots to spot any obvious deviations from normality or homoscedasticity. We assessed the influence of predictors by creating a baseline model and then added each predictor in turn; we compared each model with the previous one to verify if adding a predictor significantly reduced uncertainty. The R *Anova* function was used to compare models by performing likelihood-ratio tests between given models and report the χ^2^ value (R Core Team, 2016). We chose type 2 sum of squares or type 3 sum of squares when there were interactions to consider between predictors. *Post hoc* tests were conducted using the *glht* and *mes* functions on the complete model (R Core Team, 2016).

#### Subjective Measures

Subjective measures consisted of the answers to the experience-sampling probes. We split the 55-min sessions into four blocks of ~14 min containing eight experience-sampling probes each. We focused on task-related and task-unrelated MW frequency evolution over time and conditions using linear mixed-effect analysis. We considered blocks as a four-level categorical variable. Without specific a priori predictions regarding the block-by-block evolution, we conducted Tukey's *post-hoc* tests on the complete model.

#### Behavioral Measures

To assess performance in the auditory condition, we recorded accuracy and reaction time related to beep answers (the difference between start of the beep and the button press). The influence of attentional states and blocks on reaction time was analyzed using a linear mixed-effect analysis. We conducted Tukey's *post-hoc* tests to break the potential effects of blocks.

#### Electroencephalography

We used the ActiCHamp system and Brain Vision software (Brain Products, 2018) to record scalp potentials. A total of 64 Ag–Cl electrodes were mounted on a standard elastic cap at the standard sites of the 10–10 International system (Oostenveld and Praamstra, 2001). Impedance was kept below 5 kΩ for all electrodes. The Fpz electrode was used as the ground electrode. We used electrooculographic sites to capture eye movements. We chose the left mastoid FT9 electrode as a reference for recording.

We were interested in the influence of attentional states on stimuli perception and treatment. Beeps served as a way to measure attention through ERPs. We selected N1 (a marker of perception) and P3 (a marker of stimuli processing) elicited by the auditory task. Following the literature, we analyzed the 180–200 ms interval average on electrodes Fz, Pz, and Cz for the P3 and N1 components (Kam et al., 2011, 2014; Kam and Handy, 2013). Similarly, we chose the 380–420 ms interval average and the same electrodes for P3 component.

Regarding spectral analysis, we also used the auditory task and the time immediately preceding beeps. We focused on the upper alpha band because previous studies repeatedly revealed consistent results for the lower and upper alpha band (e.g., Benedek et al., 2011; Jauk et al., 2012). We also investigated the ASSR frequency. We chose the electrodes Pz, P1/2, P3/4, P5/6, POz, PO3/4, Oz, and O1/2 for alpha to cover the parieto-occipital region. Previous studies observed higher alpha amplitude linked with visual sensory inhibition in this region, in line with the MW perceptual decoupling (Foxe et al., 1998; O'Connell et al., 2009; Benedek et al., 2014). For the ASSR, we monitored the 39.5–40.5 Hz band where the stimulus was supposed to elicit a peak. We used the sites FCz, FC1/2 for ASSR, which had already been used by Saupe and colleagues in experiments investigating ASSR and attention (Saupe et al., 2009b; Keitel et al., 2011).

Each time an experience-sampling probe appeared, a signal was sent to the ActiCHamp software to record a trigger on the EEG signal. Similarly, another trigger was sent when participants answered the probe, whose value depended on attentional state reported by participants, and a last signal was sent by the auditory task whenever a beep played. Triggers sent by beeps served as a synchronization point to study EEG metrics, whereas triggers of probes served to classify the attentional state of participants when the beep immediately preceding played. The timing of the overall setup was tested and revealed no important deviations. We used Matlab, EEGLAB, and FieldTrip to import, re-reference, filter, epoch, remove ICA components, and build our design. The exact filtering pipeline was as follows:

- Add coordinates to existing 63 electrodes using template 10–20 location (BESA spherical format; function used: *pop_chanedit*).
- Re-reference data to FT9 and FT10 channels (Yao et al., 2005; Griskova et al., 2007; Kam et al., 2011, 2012; function used: pop_reref).
- Filter using a two-pass pass-band Butterworth filter to avoid shifting introducing the signal. The pass-band used was [0.01; 30] Hz for ERPs and [0.01; 100] Hz for ASSR and alpha (function used: *ft_preprocessing*).
- Interpolate electrodes when the line noise was deemed too important: if it displayed (1) variation above ~300 μV amplitude, (2) variation uncorrelated to other electrodes around it, and (3) previously mentioned issues were spotted on at most one subject, as the same problem found on multiple subjects would mean that the electrode itself is faulty and should be suppressed from the overall study (overall decision made after visual inspection; on average 0.1 electrode interpolated per participant for ERPs and 0.5 electrodes interpolated per participant for ASSR and alpha wave).
- Create epochs by taking signal intervals around beeps. The interval was [−800; 1,000] ms for ERPs and [−5,000; 0] ms for ASSR and alpha (on average 31.7 epochs per participant; function used: *pop_epoch*).
- Remove the baseline of each epoch: for ERPs, we took the average signal in [−200; 0] ms and subtract it from the whole epoch; for ASSR and alpha, remove base power of each frequency (function used: *pop_rmbase*).
- Discard epochs when they were heavily contaminated by muscle artifacts which would lower ICA power (decision made after visual inspection, although multiple backs and forth were made to determine ICA impact tolerance; on average 3.3 epochs discarded per participant for ERPs, 4.4 epochs discarded per participant for ASSR and alpha wave; function used: *eegplot*).
- Run the ICA with option “extended, 1” also reducing the number of dimension by one due the rank deficient matrix (function used: *pop_runica*).
- Discard components in case of ocular movements (high power coupled with activity frontal, dissymmetrical from both eyes perspective, spatially and temporarily narrowed), blinks (high power coupled with activity frontal, symmetrical from both eyes' perspective, spatially and temporarily narrowed), other muscle activity (very high power coupled with activity spatially and temporarily narrowed), and electrode malfunction (very high power, activity centered on one specific electrode). The final decision was made after visual inspection (no epochs discarded for ERPs, on average 1.6 epochs discarded per participant for ASSR and alpha wave; function used: *pop_selectcomps*).

We then exported data to R to perform statistical analysis. We used a linear mixed-effect analysis to look at the influence of attentional states on ERPs, alpha, and ASSR amplitude.

## Results

### MW Frequency Analysis

**Figure 3:**
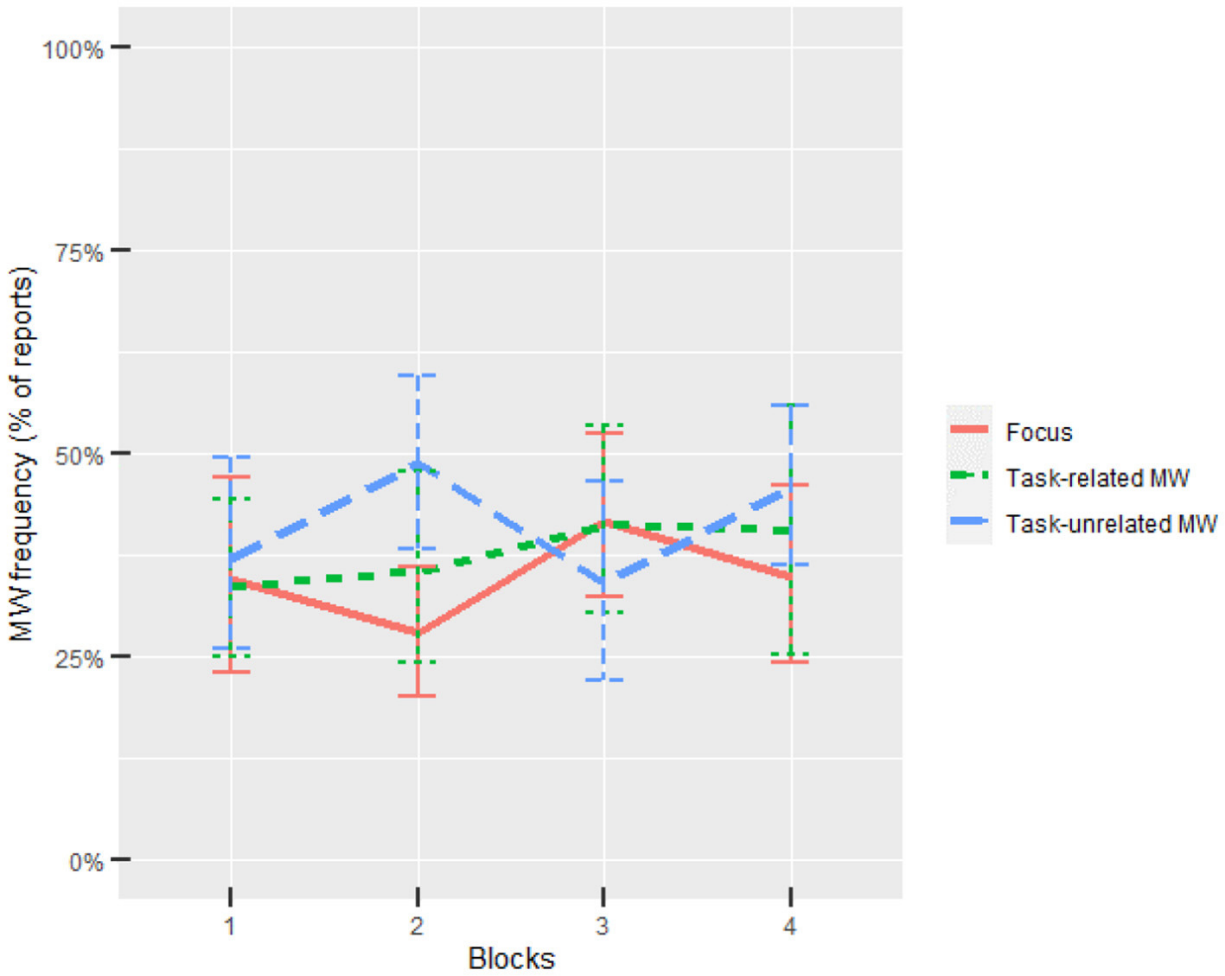
Task-related and task-unrelated MW evolution through blocks. Error bars show the 95% CIs based on bootstrap.

Participants reported on average 31.3% task-related MW (*SD* = 4.4%) and 36.6% task-unrelated MW (*SD* = 5.0%, see Figure 3, Supplementary Data Sheet 1). This rate is consistent with previous studies (Smallwood et al., 2006; Smallwood and Schooler, 2015; Gouraud et al., 2018a,b). Each participant reported on average 1.5% “External distraction” reports (*SD* = 1.21). Considering this low rate, we discarded “External distraction” reports and adopted the ternary approximation of attentional states (i.e., either focused, task-related MW, or task-unrelated MW). All participants answered all 32 probes, except one participant who did not answer four probes.

Blocks did not significantly influence task-related MW. On the contrary, blocks significantly influenced task-unrelated MW rates, χ^2^ = 12.13, *p* = 0.007. *Post-hoc* tests revealed that task-unrelated MW rate were significantly higher under the second block compared with the first and third blocks, *p* = 0.021, *d* = 0.55, *p* = 0.010, *d* = 0.62, respectively. All results from model comparisons are gathered in Table 1, bold values being significant.

**Table 1:** Influence of blocks on task-related and unrelated MW frequency.

		**Task-related MW**		**Task-unrelated MW**	
**Effect added**	***df***	**χ*^2^***	***p*-value**	**χ*^2^***	***p*-value**
Block	3	0.30	0.828	**12.13**	**0.007**

*Bold values are significant results*.

### Auditory Task: Reaction Time to Beeps

**Figure 4:**
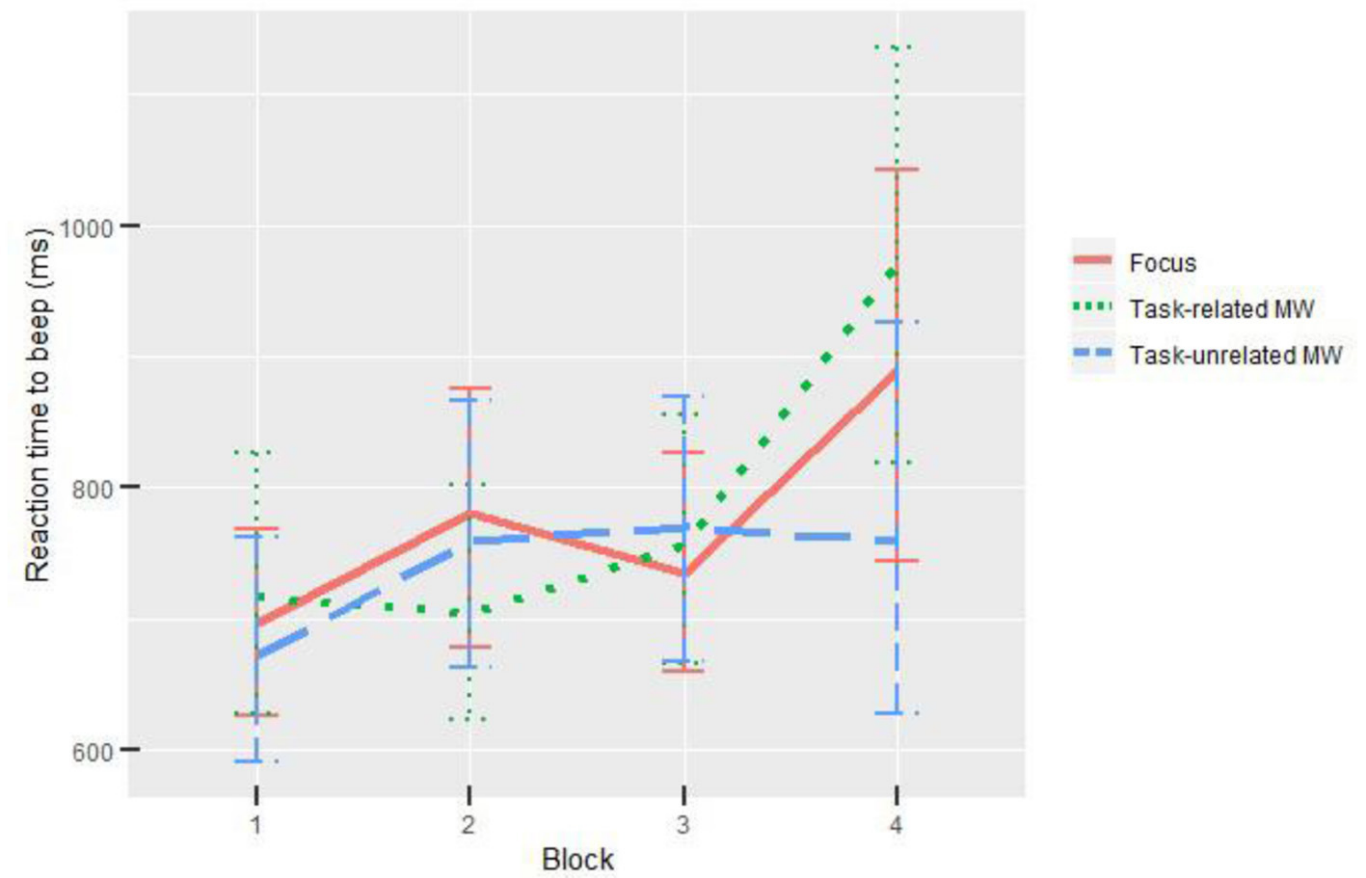
Influence of blocks and attentional states on beep reaction time. Error bars show the 95% CIs based on bootstrap.

The auditory task performance was investigated using reaction time when presented a beep followed by a probe. Participants reacted to on average 31.3 beeps out of the 32 presented. Attentional states did not influence reaction time. On the contrary, there was a significant influence of blocks on reaction time, χ^*2*^ = 25.52, *p* < 0.001. *Post-hoc* tests revealed that participants were significantly slower during the fourth block compared with the first and third blocks, respectively (*p* = 0.007, *d* = 0.48 and *p* = 0.016, *d* = 0.28). All results from model comparisons are gathered in Table 2 and illustrated in Figure 4.

**Table 2:** Influence of attentional states and blocks on beep reaction time.

**Effect added**	***df***	**χ^*2*^**	***p*-value**
Attentional states	2	2.89	0.24
Block	3	**25.52**	**<0.001**
Attentional states: blocks	6	10.09	0.121

*Bold values are significant results*.

### Auditory Task: Influence of Attentional States on ERPs

**Figure 5:**
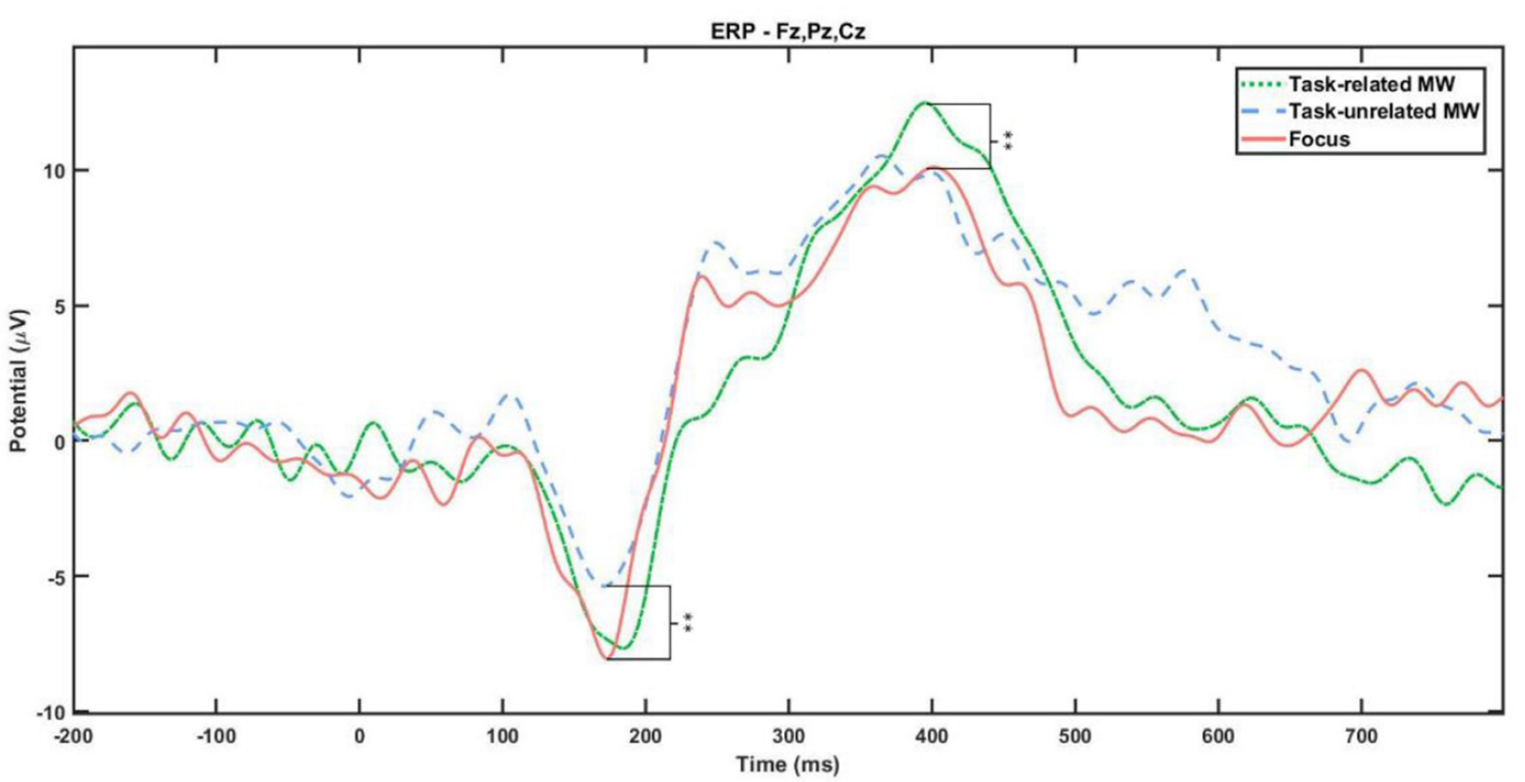
Beep ERP signal for task-related MW (green), task-unrelated MW (blue), and focus (red) attentional states.

The amplitude evolution of ERPs elicited by the auditory task (beeps) was investigated. Attentional states significantly influenced both N1 and P3 components (see Table 3 and Figure 5). *Post-hoc* tests revealed that for the N1 component, reports of task-unrelated MW were accompanied with a lower amplitude (*M* = −6.06 μV, *95% CI* = [−8.01; −4.12] μV) compared with periods of focus (*M* = −9.39 μV, *95% CI* = [−12.21; −6.60] μV), *p* = 0.024, *d* = 0.36. For the P3 component, the statistics showed a significantly higher amplitude for task-related MW (*M* = 12.69 μV, *95% CI* = [9.28; 16.13] μV) compared with focus periods (*M* = 8.20 μV, *95% CI* = [5.54; 10.85] μV), *p* = 0.009, *d* = 0.16.

**Table 3:** Influence of attentional states on the amplitude of the ERP components N1 and P3.

**Effect added**	***df***	**N1 component**		**P3 component**	
		**χ^*2*^**	***p*-value**	**χ^*2*^**	***p*-value**
Attentional states	2	**9.41**	**0.009**	**8.83**	**0.012**

*Bold values are significant results*.

### Visual Task: Influence of Attentional States on Alpha Wave Amplitude

**Figure 6:**
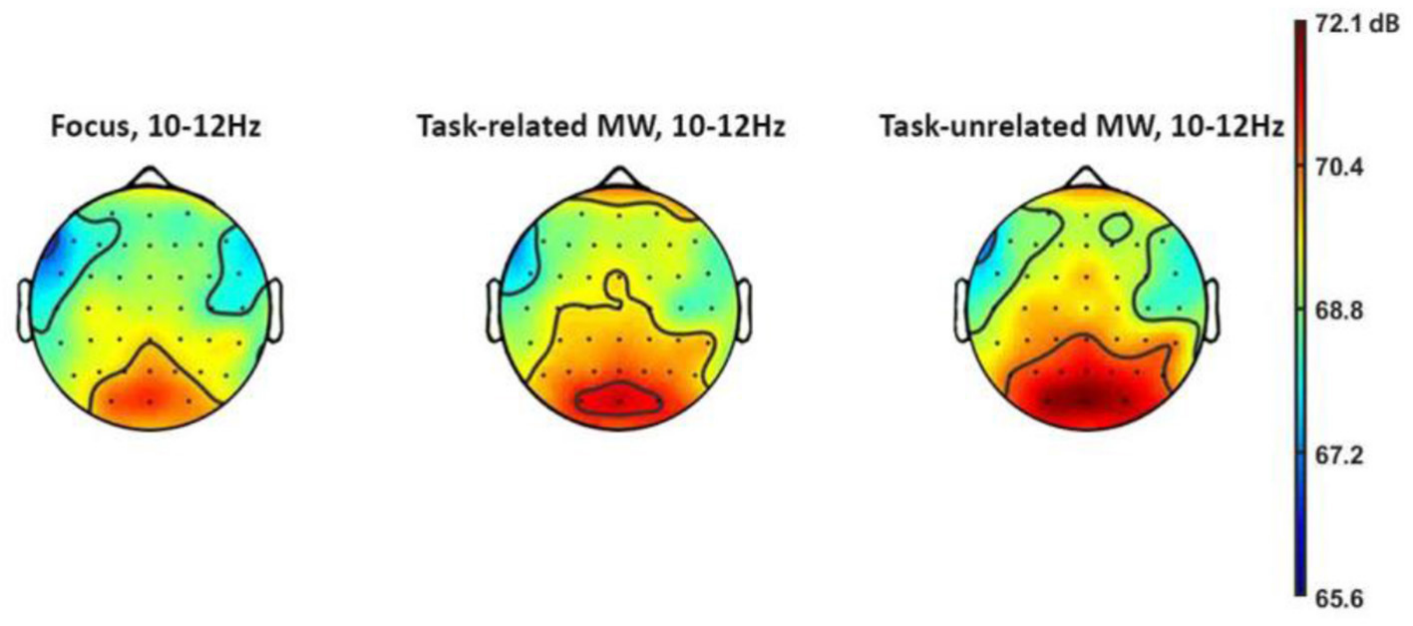
Topography of alpha frequency for each attentional state.

Alpha wave power evolution before experience-sampling probes was investigated. Results showed a significant influence of attentional states on alpha amplitude (see Figure 6 and Table 4, bold values being significant), χ^2^ = 8.35, *p* = 0.015. *Post-hoc* tests showed significantly higher alpha amplitude during task-unrelated MW (*M* = 53.83 μV^2^/Hz, *95% CI* = [52.35; 55.31] μV^2^/Hz) compared with focus episodes (*M* = 53.03 μV^2^/Hz, *95% CI* = [51.90; 54.16] μV^2^/Hz), *p* = 0.014, *d* = 0.27. Other comparisons (task-related MW vs. focus, task-related MW vs. task-unrelated MW) were not significant.

**Table 4:** Influence of attentional states on alpha and ASSR amplitude.

**Effect added**	***df***	**Alpha power (log)**		**ASSR amplitude**	
		**χ^2^**	***p*-value**	**χ^2^**	***p*-value**
Attentional states	2	**8.35**	**0.015**	2.55	0.279

*Bold values are significant results*.

### Influence of Attentional States on ASSR Amplitude

**Figure 7:**
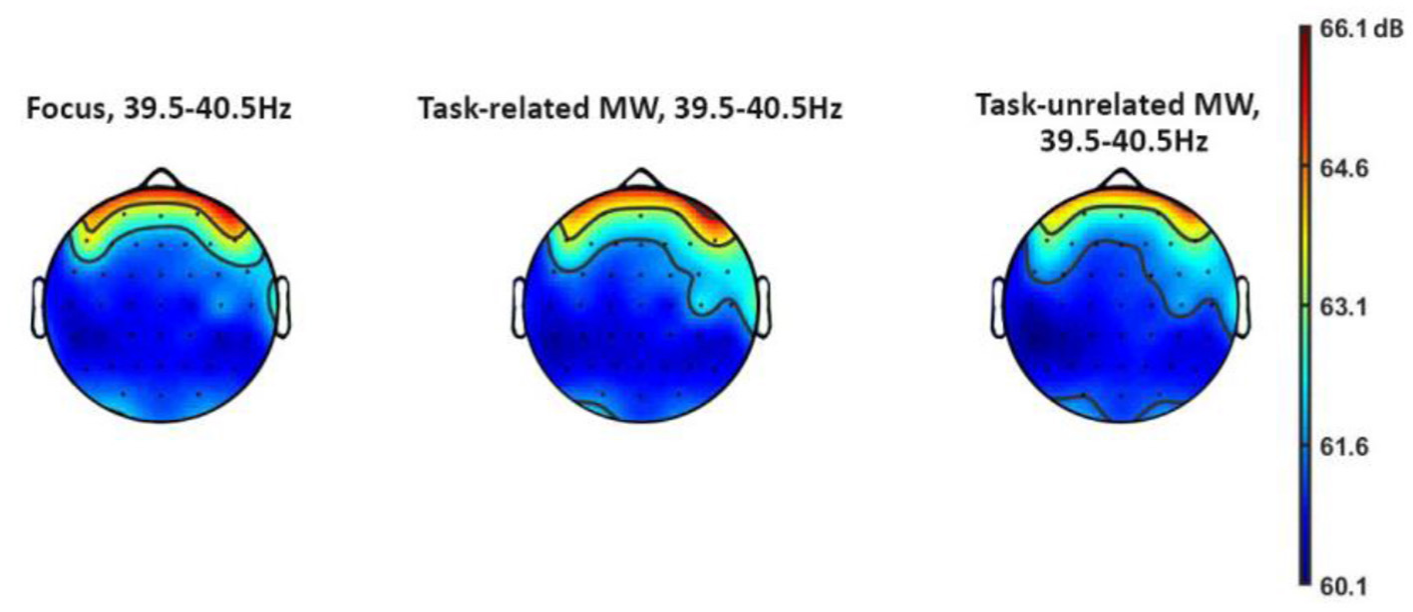
Topography of ASSR frequency for each attentional state.

**Figure 8:**
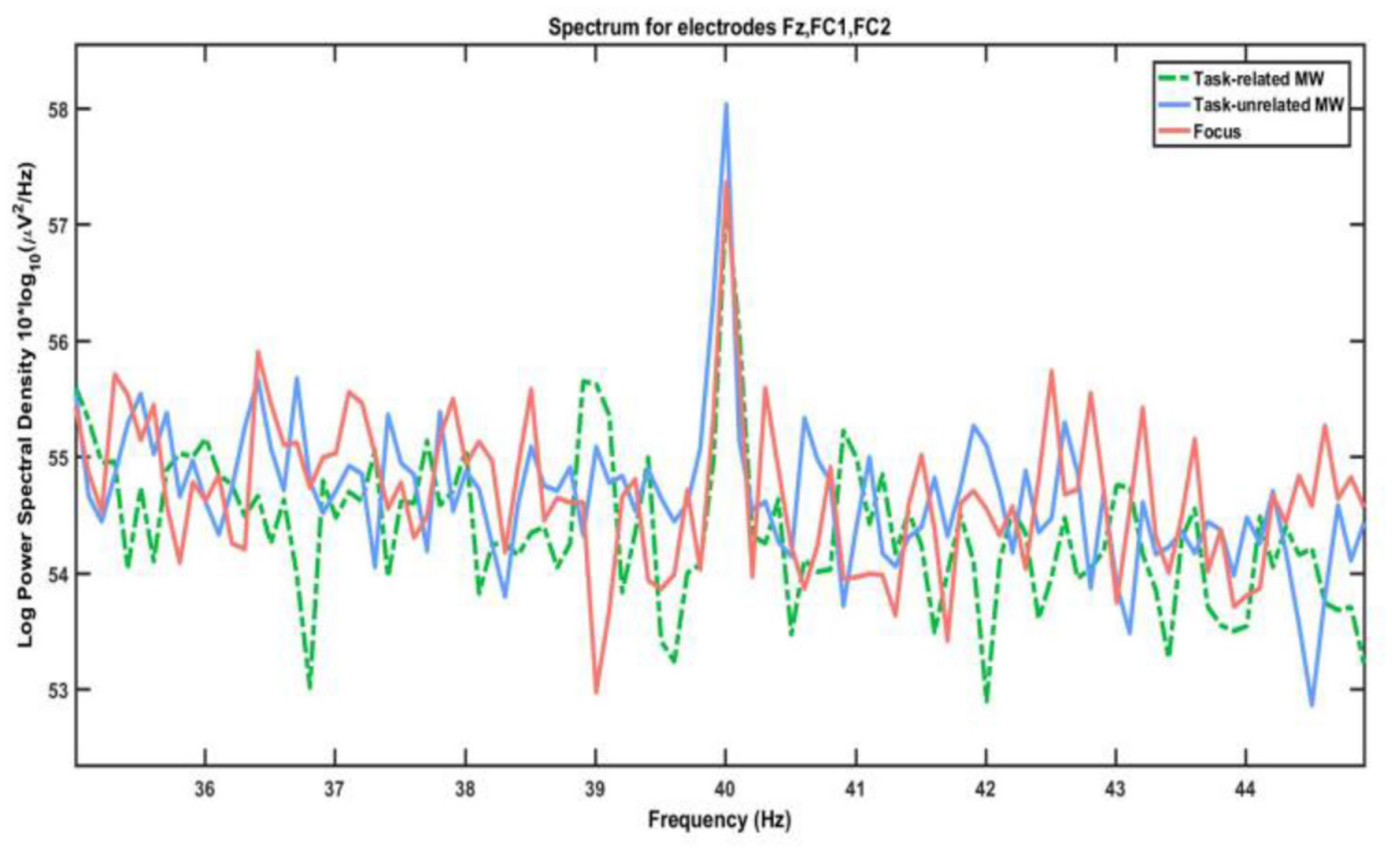
Spectrum of 35–45 Hz interval for each attentional state.

**Figure 9:**
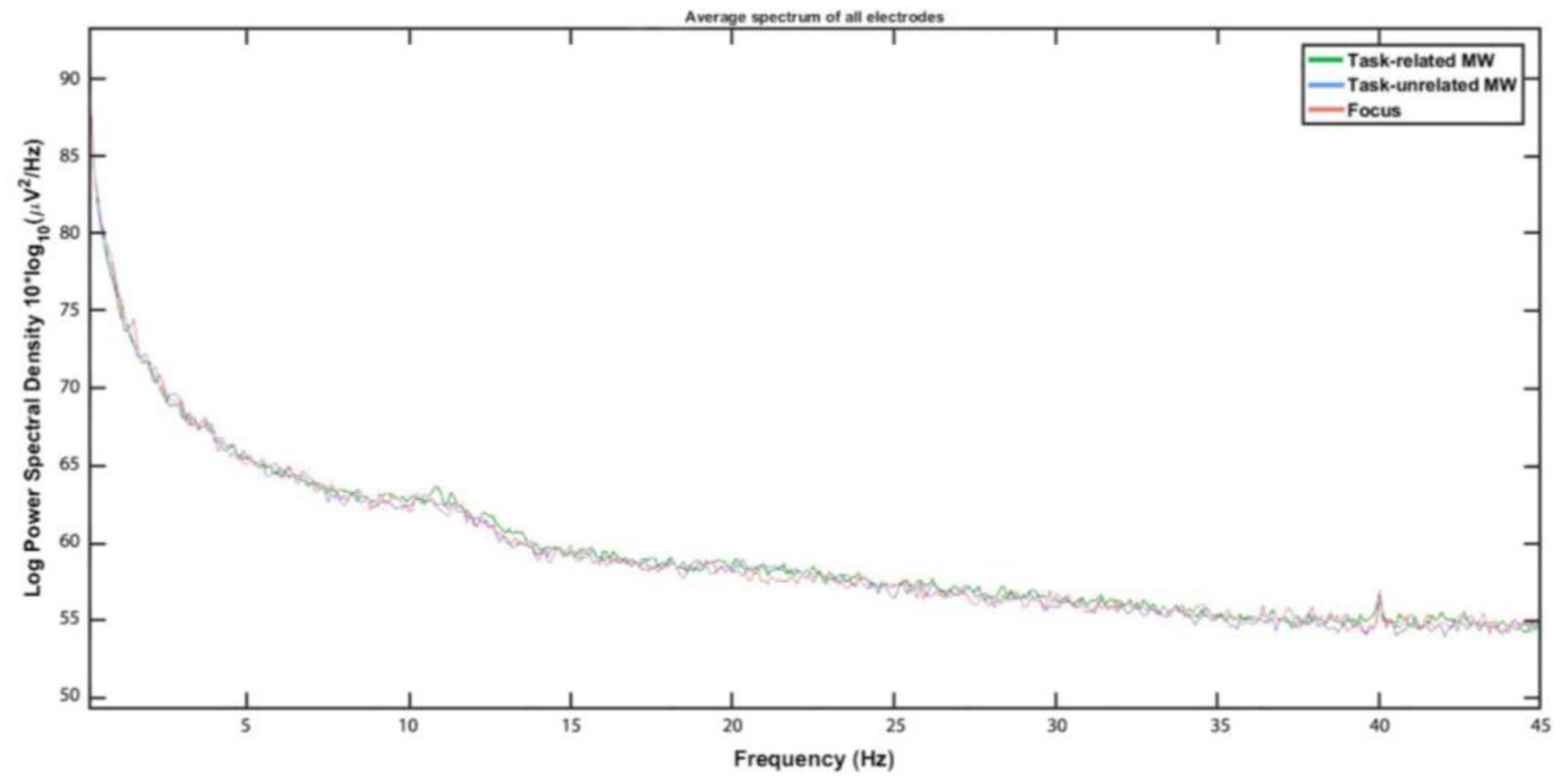
Spectrum of 0–45 Hz interval for each attentional state.

No influence of attentional states on ASSR amplitude was uncovered (Figure 7). However, spectral plots still revealed a peak at 40 Hz, showing that the ASSR was visible on participants' spectrum even during this complex task (see Figures 8, 9). Should anyone want to reuse this background noise for other ASSR activities within aeronautical-inspired environments, we mention that 12 participants out of 18 reported that they felt the noise was similar to a propeller airplane.

## Discussion

The aim of this study was to evaluate the viability of MW neuronal markers in complex ecological automated environments, and to help characterize features of the attentional decoupling in these settings. We chose an automated obstacle avoidance task that participants had to supervise while reacting as fast as possible to beeps they heard. EEG signal was chosen to acquire cerebral activity in the form of ERPs, alpha wave amplitude, and ASSR. To yield detailed results, we decomposed MW into task-related and task-unrelated acquired using attentional probes. We decomposed the 40-min task into 4 blocks of 10 min each. Participants did not show any increase in task-related or non–task-related MW during the time spent on the task although more task-unrelated MW emerged during the second block. When analyzing ERP components created by beeps, we observed lower N1 component amplitude during task-unrelated MW, while P3 component had higher amplitude during task-related MW, compared with other attentional states. Alpha wave activity was higher in parieto-occipital regions during task-unrelated MW compared with other attentional states. Finally, ASSR was clearly elicited, but its amplitude was not significantly influenced by attentional states. Overall, these results underline the complex influence of the MW perceptual decoupling on operator's behavior in ecological environments and have several implications when considered together.

### Measuring the Impact of MW

Taken together, the observed effects support a reduction in cortical processing of the external environment during task-unrelated MW. First, for the auditory task, N1 component elicited by the beeps had a lower amplitude during task-unrelated MW, indicating a state of reduced perception of stimuli already identified by Kam et al. (2011). Participants who experienced task-unrelated MW were less receptive to the beeps. Nevertheless, only a non-significant trend could be observed in reaction times (Figure 4), with subjects being faster during the fourth block for task-unrelated MW compared with other attentional states. Subjects may have focused, maybe even attention-tunneling, on the visual task when being focused or in task-related MW. On the contrary, being in task-unrelated MW may have led participants to use strategies favoring speed over precision, without significant impact on the accuracy due to the low difficulty of the task (Salomone et al., 2021).

Second, regarding the visual task, the increase in alpha power in the parieto-occipital lobe shows that participants inhibited visual perception during MW episodes (Foxe and Snyder, 2011; Benedek et al., 2014; Clayton et al., 2015). Although the debate still exists on alpha power, both analyses are congruent and consistent with research sharing the same features, i.e., probe-caught MW (Baird et al., 2014), visual (Compton et al., 2019), and ecological task (Baldwin et al., 2017). MW creates a decoupling from the task at hand, even in complex bimodal environments. Our results are a first step toward filling the gap between real consequences of MW (Galera et al., 2012; Berthié et al., 2015) and EEG research in laboratory settings (Kam, 2010; Kam et al., 2019). Taken together, visual and auditory analyses support the multimodal influence of MW in complex environments (Kam et al., 2011), although our setup does not allow us to make quantified claims and compare modalities.

We observed no effect of attention on ASSR amplitude, even though its evoked power was visible on the EEG spectrum of the participants. This outcome is in line with the results of O'Connell et al. (2009) regarding the absence of amplitude modulation of MW on SSR. It is possible that our experiment did not succeed because of its features, such as the use of amplitude modulation instead of clicks (Voicikas et al., 2016) or the insufficient number of participants. Another possibility may be that SSR produced by non-target background noise is already being reduced by participants instructed to ignore it from the start; it may therefore not be further influenced by MW. However, this hypothesis is in contradiction with both literature on ASSR in attention modulation settings (Skosnik et al., 2007; Müller et al., 2009; Mahajan et al., 2014) and our own results regarding lower N1 amplitude during task-unrelated MW. To account for this observation, a final explanation may be that internally directed attention like MW is fundamentally different from the evolution of external direction between sensory modalities. In this case, the absence of amplitude modulation would show that MW does not impact the earliest stages of perception, allowing for a basic processing of external stimuli. Further work in this area is needed to provide robust conclusions.

### Gradual Impact of MW

Important differences were highlighted between task-related and task-unrelated MW, supporting the existence of “depth” or “intensity” (related to the decoupling) in MW episodes. During task-unrelated MW, participants inhibited perception of auditory stimuli (as shown by the N1 amplitude), but not during task-related MW compared with focus moments. On the contrary, auditory information processing (P3 amplitude) was higher during task-related MW than during focus intervals. Participants reporting being focused may actually focus on the visual part of the task (the most cognitively demanding) while inhibiting all auditory stimuli, whether relevant to the task or not. On the other hand, task-related MW may create a more superficial decoupling than task-unrelated MW. This mental state may redirect attentional resources from the exhausting visual task to listening to auditory cues, thus participating in a more balanced resource allocation independently of task demand. Unfortunately, we did not observe differences in performance, i.e., reaction time during the auditory task. It is likely that because the processing of auditory stimuli did not require much cognitive resources, superficial perception was enough to perform it.

Previous explanation remains very conditional, as the available observations are not sufficient to definitely establish the depth of MW. A graded MW with a different decoupling could explain why we are most of the time able to perform tasks while being in MW, while sometimes we make clear errors that could have been avoided with our full attention (Cheyne et al., 2006; Carriere et al., 2008; Farley et al., 2013). Two protocols may complete the present study in relation to MW depth: using the same experiment, but asking the participant to ignore the beeps; the irrelevance of beeps may thwart interesting results when analyzing the influence of task-related MW on ERPs. Another possibility would be to use the same experiment once again, but this time participants would have two different beeps to react to, each associated with a different button. The needs for more processing of auditory stimuli could link the performance data to MW decoupling depth. Nevertheless, more data are needed to rule over the depth dimension.

#### Factors Stimulating MW Emergence

In this experiment, MW rates remained mostly stable through time-on-task, only the second block exhibiting higher task-unrelated MW rates compared with the first and third ones. We witnessed similar behavior in our previous study (although here MW increased in the middle of the task instead of decreasing, see Gouraud et al., 2018a). Literature generally agrees that MW rates should increase with time-on-task (Smallwood et al., 2002; Pattyn et al., 2008; Risko et al., 2012; Gouraud et al., 2018a) although several studies failed to observe such behavior (Thomson et al., 2014; Arnau et al., 2020). Nevertheless, the exact link between MW and time-on-task may be mediated by task difficulty, i.e., task demands in attentional resources (McVay and Kane, 2009; Krimsky et al., 2017). We have already used as the only task our automated UAV monitoring environment in previous experiments without observing more MW, which shows that the multitasking did not require much attention from participants (see Mind Wandering Frequency Analysis and Gouraud et al., 2018a,b). Moreover, attention demand remained constant throughout the task, which further decreased the possibility of bias in our subsequent analysis. To explain the lack of increase in MW with time-on-task, a first explanation might be that participants, aware of the overall duration of the experience, sensed time passing by and reengaged in the task in the second half (Arnau et al., 2020). The lack of MW increase with time-on-task might also be due to automation errors, placed at the ends of the second and third blocks. A third possibility might be explained by a too disruptive setup (e.g., beeps allowing reengagement, EEG being too uncomfortable). However, our previous experiments with the same visual environment, but no auditory stimuli, yielded equivalent attentional state percentages on average (Gouraud et al., 2018a,b).

More generally, the question of what conditions will stimulate the emergence of MW remains, both in experiments and in the open. Time-on-task plays an important role (Smallwood et al., 2002). However, it may not be the only factor: on top of various individual features linked with different MW rates [training in Casner and Schooler (2015); positivity in Hancock (2013); gender in Mar et al. (2012); creativity in Zedelius and Schooler (2016)], the very nature of tasks to perform could influence MW and its evolution. In particular, operators faced with increased automation see their relation to the task dramatically modified. We already investigated the influence of automation levels in a previous experiment (Gouraud et al., 2018b) without significant differences in MW rates between a manual and an automated condition.

Nevertheless, many dimensions of automation that could influence MW rates remain unexplored. One of the main impacts of higher automation is a drop in operators' sense of control or agency (Haggard, 2017). Sense of agency is the experience of identifying oneself as the author of an action and its consequences (Gallagher, 2000). This form of self-awareness is important not only for motor control but also for causal responsibility and serves as a key motivational force for human behavior. Recently, it has been shown that the sense of agency could be dramatically impaired when interacting with automation (Berberian, 2019). While co-workers develop a form of we-agency (Crivelli and Balconi, 2010; Obhi and Hall, 2011), the same does not stand true for human–system cooperation (Wohlschläger et al., 2003a,b; Glasauer et al., 2010; Sahaï et al., 2017). Similarly, there is a loss of agency when operators' tasks shift from working a system to monitoring it (Berberian et al., 2012). Even though automation generally brought safer and more productive systems, the loss of agency could generate task disengagement and be one of the main reasons why operators are unable to regain manual control in critical situations (Bainbridge, 1983; Endsley and Kiris, 1995; Cummings, 2004; Louw et al., 2015b; Berberian et al., 2017). Critically, Wen and Haggard (2018) have highlighted important differences in attention allocation correlated with differences in the sense of agency: the loss of a sense of control could decrease the allocation of attentional resources to stimuli relevant to the task at hand. In this context, loss of agency may have a significant influence on MW rates. To our knowledge, no experiment has investigated the relation between MW and agency.

#### MW and Operator Engagement Issue

As our results showed, distinguishing different types of MW revealed different impacts on EEG measures, while the absence of MW influence on ASSR may highlight a fundamental difference between internally and externally directed attention. Despite these unknowns, our results add to the existing literature supporting the decoupling hypothesis and linking MW to a form of attentional disengagement. Indeed, task engagement strongly modulates performance through goals and motivation (Bedny and Karwowski, 2004; Fairclough et al., 2013; Leontiev, 2014), concepts that are strongly linked with MW (Cheyne et al., 2009; Danckert, 2017; Gouraud et al., 2018b). MW could exacerbate task disengagement by highlighting the discrepancy between entertaining thoughts and the ungratifying present (Smallwood and Schooler, 2006; Eastwood et al., 2012) and drawing attention to one's own failure to maintain vigilance (Critcher and Gilovich, 2010; Westgate and Wilson, 2018). Other researchers believe that MW may be just a symptom of boredom: internal sources of stimulation could serve as a second-best option when external tasks fail to keep us focused (Singer, 1975; Bench and Lench, 2013). Neurologically, MW episodes are characterized by the deactivation of the dorsolateral prefrontal cortex (DLPFC, see Christoff et al., 2009; Stawarczyk et al., 2011). DLPFC interacts with dorsal and ventral attentional pathways to shift and focus attention on the most relevant stream of task-related information (Johnson and Zatorre, 2006). It is a network thought to play a crucial role in maintaining task engagement (Curtis and D'Esposito, 2003). MW is thought to represent the lower end of a continuum of task engagement (Lee, 2014; Dehais et al., 2020).

MW pertains to a wider collection of mental states linked to engagement and negatively impacting performance. These suboptimal neurocognitive states are investigated by neuroergonomics, whose purpose is the study of the human brain in relation to performance at work and in everyday settings (Parasuraman, 2011; Gramann et al., 2017). The development of this field has been facilitated by the twenty-first century revolution in our understanding of neural mechanisms, but also by recent developments in advanced and portable neuroimaging techniques (Dehais et al., 2020). Several attempts have been made to identify MW features within dry EEG signals, with success on ERPs and alpha waves (van der Wal and Irrmischer, 2015; Kam et al., 2019). Functional Neuro InfraRed Spectroscopy (fNIRS) has also demonstrated its capability to detect MW episodes in ecological simulation by monitoring the Default Mode Network (Durantin et al., 2015), a network involved in attention drifting processes (Raichle et al., 2001; Konishi et al., 2015; Golchert et al., 2016). Both dry EEG and fNIRS could be integrated into operational environments with little disruption for the user (Mullen et al., 2015; OpenBCI, 2016; This Place, 2016; SmartCap, 2020). On top of neuroimaging techniques, oculometry has also been substantially improved over the past decade, producing efficient, small, and cheap devices. It has demonstrated a high sensitivity to MW in safety-critical environments, although only in simulators (Louw et al., 2015a; Louw and Merat, 2017). Thanks to these systems and models, neuroergonomics could help translate MW findings from psychology and neurosciences into procedures changes to enhance safety in the industry.

## Conclusion

We presented the results of an EEG study with a visual (monitoring and correction of an automated UAV avoiding obstacles) and an auditory (infrequent beep which required fast button press) task presented simultaneously with the aim to understand the cerebral signature of MW. Participants also heard a background noise designed to elicit ASSR. We saw that task-related and task-unrelated MW exhibit a different EEG signature, whether it is on ERP components or on alpha waves, suggesting the existence of depth in perceptual decoupling. Our results also stress the need to carefully discriminate MW dimensions when evaluating MW-induced decoupling. Finally, the absence of MW hallmark on ASSR amplitude does not support the possibility to use SSR to study MW continuously. However, it also means that the earliest stages of perception may not be impacted by attentional decoupling.

Overall, our results highlight the crucial need to study the neural correlates of MW to identify its exact influence on operators. Even though the setup involved remained highly controlled and laboratory related, our tasks were relatively close to complex automated environments encountered in operations, and more specifically teleoperations. Contrary to recent claims (Neigel et al., 2019), MW pervasive effects have been widely reported in monotonous ecological simulations (He et al., 2011; Casner and Schooler, 2014, 2015; Louw et al., 2015a,b; Baldwin et al., 2017; Gouraud et al., 2018a,b) and real environments (Galera et al., 2012; Berthié et al., 2015). Moreover, they are perfectly integrated in several recent neuroscientific models (Pattyn et al., 2008; Dehais et al., 2020). Other problems teleoperations should overcome involve operators' ability to mentally jump into a situation while being physically away and should be specifically assessed, and the related issues studied. Distraction and other forms of inattention are already a significant safety problem within the transport industry, e.g., in the air (Loukopoulos and Field, 2001; Casner and Schooler, 2015) or on the road (Galera et al., 2012; Berthié et al., 2015). In this context, a better understanding of MW, which participates in operator distraction, is crucial to limit distraction consequences. It is essential that research investigates the effects of the different characteristics of MW, while the possibilities to mitigate its consequences must also be examined through both ecological setup and operational environments and the outcomes adopted by the industry. Taking the problem into account when designing the technology (Nielsen et al., 2007; Hosseini and Lienkamp, 2016) could enhance teleoperations and install it as the next important step toward full automation. In this context, neuroergonomics could bring a new perspective on this kind of suboptimal neurocognitive state to go further than broad metaphorical concepts.

## Data Availability Statement

The raw data supporting the conclusions of this article will be made available by the authors, without undue reservation.

## Ethics Statement

The studies involving human participants were reviewed and approved by ONERA ethical committee. The patients/participants provided their written informed consent to participate in this study.

## Author Contributions

All authors listed have made a substantial, direct and intellectual contribution to the work, and approved it for publication.

## Conflict of Interest

The authors declare that the research was conducted in the absence of any commercial or financial relationships that could be construed as a potential conflict of interest.
